# Macular Hole in Myopic Eyes: A Narrative Review of the Current Surgical Techniques

**DOI:** 10.1155/2019/3230695

**Published:** 2019-03-11

**Authors:** Chiara De Giacinto, Marco R. Pastore, Gabriella Cirigliano, Daniele Tognetto

**Affiliations:** Eye Clinic, Department of Medicine, Surgery and Health Sciences, University of Trieste, Trieste, Italy

## Abstract

Macular hole (MH) in myopic eyes is a disease arising from complex tractional forces exerted by vitreomacular interface, epiretinal tissue, and progressive scleral ectasia of the posterior ocular globe wall. This retinal disease requires vitreoretinal treatment for its repair, and the surgical intervention remains a challenge also for experienced surgeons. The aim of this review is to describe the current knowledge regarding the pathogenesis of MH in myopic eyes and to detail novel surgical techniques and technological advancements in its surgical management.

## 1. Introduction

Idiopathic macular hole (MH) is an important cause of central visual loss [1] characterized by a round full-thickness defect in the center of the macular area involving the anatomical fovea [2, 3]. The estimated prevalence in the general population is approximately 3.3 per 1000 people [4, 5], with a cumulative incidence of MH formation of 41.1 cases per 100 000 person/years [6]. This incidence increases with age, and the disease usually affects individuals in their sixth or seventh decade of life [7]. Females have a 64% increased risk of being diagnosed with MH when compared with males [6]. MH is a common complication in pathological myopic eyes with an axial length greater than 26.5 mm and/or a refraction greater than −6.00 diopters [8, 9], and a prevalence of 8.4% has been reported [10]. The incidence of myopic MH is greater in Asian ethnicity compared to Caucasian or black individuals due to the high prevalence of myopic refractive defect in Asian population [6].

For many years, MH was considered an untreatable disease resulting in irreversible vision loss. In the early 90s, new surgical strategies to treat this anatomical macular defect in order to improve the visual acuity were described. The surgical procedure consisted of three-port pars plana vitrectomy (PPV) with posterior hyaloid removal in combination with intraocular gas tamponade to allow the flattening and the repositioning of MH edges [11, 12]. A remarkable success rate and a clear benefit from this type of surgical management were reported [13–16].

Although pars plana vitrectomy with posterior hyaloid removal, internal limiting membrane (ILM) peeling, long-acting gas tamponade, and face-down positioning is still the gold standard for a successful closure of idiopathic MH, the treatment of large, persistent, or myopic MH remains a challenge [17–19]. Nowadays, several methods have been proposed to allow high rate of success, particularly for the abovementioned MH such as the inverted ILM flap [20], inverted ILM flap insertion [21] with or without blood coverage of the hole [22], temporal or superior single-layer inverted ILM flap [23, 24], free ILM insertion [25], and double [26] or multiple free ILM flap insertion [16] into the MH.

The purpose of this review is to describe the current knowledge regarding the pathogenesis of MH in myopic eyes and to detail novel surgical techniques and technological advancements in the surgical management of this retinal disease.

## 2. Pathogenesis

The biomicroscopic identification of MH in highly myopic eyes may be difficult due to several factors such as the typical low contrast between the anatomical foveal defect and the retinal pigmented epithelium (RPE), the chorioretinal atrophy, or the presence of a deep posterior staphyloma [27].

The introduction of optical coherence tomography (OCT), which provides an in vivo analysis of the relationship between the macula and the vitreous, has allowed identifying the perifoveal vitreous changes that may lead to MH formation. In particular, OCT is able to detect the localized adhesion between vitreous cortex and fovea, the incomplete posterior vitreous detachment associated with a full-thickness MH [28–30] and the ILM contraction that creates a tangential traction responsible of MH enlargement [19].

The anteroposterior traction caused by vitreous cortex on the fovea leads to MH formation [28–30]. It is widely accepted that the genesis of this dynamic force is localized in the precortical liquified vitreous pocket, immediately in front of the macula area, resulting in a passive contraction of the posterior hyaloid undetached from the fovea [31, 32]. Subsequently, the tangential traction due to the ILM stiffening promotes the progressive MH enlargement.

**Figure 1:**
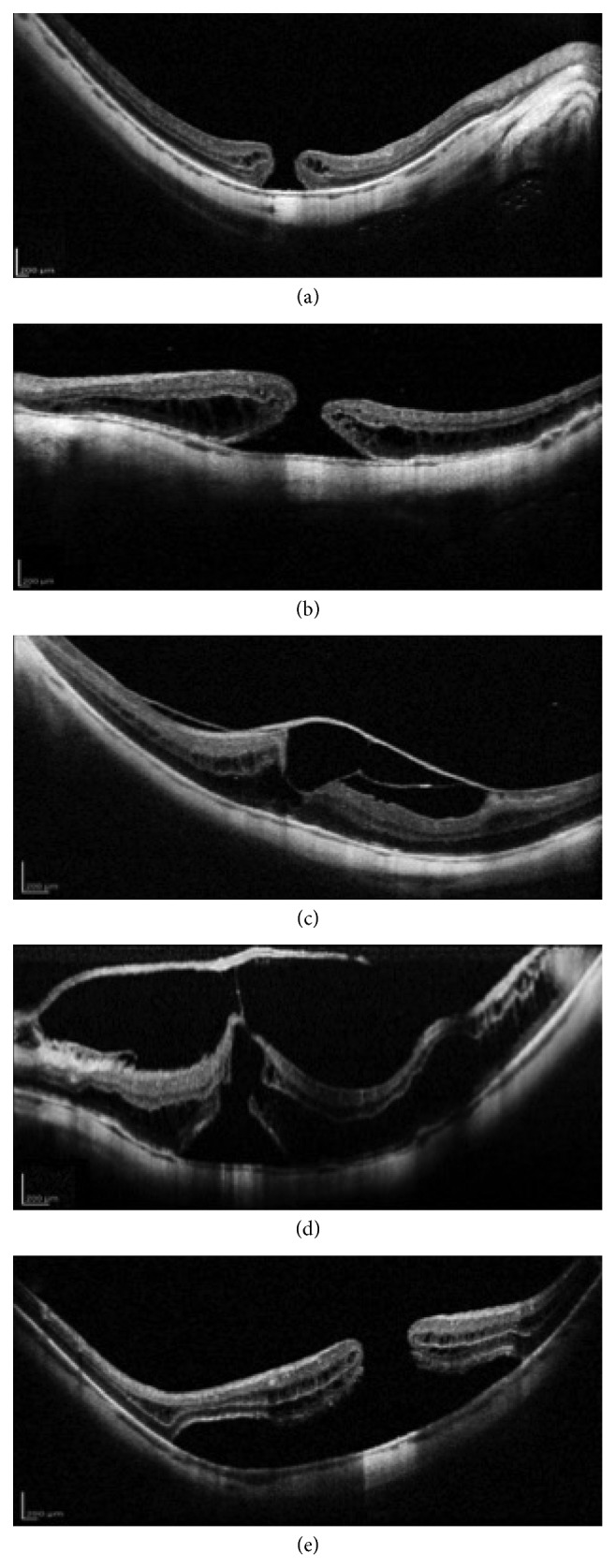
Different myopic macular hole (MH) patterns. (a) Full-thickness MH with intraretinal cysts in deep posterior staphyloma. (b) Full-thickness MH with perilesional foveoschisis. (c) Vitreomacular separation with vitreofoveal adhesion which causes the dehiscence of the inner retinal layers and the formation of inner foveal cysts. (d) Vitreomacular traction with impending macular hole associated with diffuse macular schisis and foveal detachment which results in outer retinal layers disruption. Note: the important traction exerted by the thickened posterior hyaloid undetached from the fovea and the consequential retinal split due to the deep posterior staphyloma. (e) Complete disruption of the inner and outer retinal layers resulting in macular retinal detachment.

In contrast, in high myopic eyes, MH formation is related to more complex pathogenetic mechanisms [33–38]. In addition to the anteroposterior traction caused by the strong adhesion of vitreous cortex and to the tangential traction from the tightening of the ILM, an important role is reserved to the abnormal growth of myopic eyes that may result in the development of posterior staphyloma (PS) defined as a progressive scleral ectasia of the posterior ocular globe wall (Figure 1(a)) [39].

The PS introduces new pathogenetic forces in myopic MH formation and increases the spectrum of tractional forces in relation to different depths and location of the scleral ectasia with respect to the macular area. The centripetal vector forces are more relevant in case of deeper PS compared to the flatter staphyloma [40]. Due to the relative inelasticity of the inner retinal layers and retinal vessels, deep PS promotes a progressive retinal split which initially results in the development of a myopic foveoschisis (FS) (Figure 1(b)) and finally may lead to a retinal detachment involving the posterior pole.

In the case of flat PS, vitreoretinal tractions are not sufficient to produce the concomitant FS or retinal detachment [41, 42], and the pathogenetic mechanism of MH formation seems to be similar to those of emmetropic eyes [43].

Recently, several mechanisms regarding the development of MH in highly myopic patients were analyzed, and different pathways of MH formation were reported [44]. In detail, a normal foveal depression with abnormal vitreoretinal relationship characterized the first pathway. According to the main tractional forces recognized, two different processes can be distinguished. Similar to MH in emmetropic eyes, in high myopic eyes, the anteroposterior vitreomacular traction (VMT) induces a vitreomacular separation with the disruption of the inner laminar layers and the formation of inner foveal cysts with or without small foveal detachment (Figure 1(c)). The rupture of intraretinal cysts results in a disruption of outer laminar layers followed by the progression in a full-thickness MH. Alternatively, the posterior hyaloid may separate from the fovea, and the development of a tangential traction exerted from the surrounding preretinal membrane leads to a lamellar MH formation. The progressive foveal disruption results in a full-thickness MH [44].

A second pathway is characterized by the presence of FS that may occur with or without macular detachment. FS is not an uncommon finding in highly myopic eyes (9–34%) [43] and is believed to be one of the main causes of MH formation in these patients [45–47]. A macular schisis promotes a tangential or multidirectional traction with the development of lamellar MH. The continuous traction caused by the preretinal membrane induces the evolution in a full-thickness MH [44]. When macular schisis occurs with foveal detachment, an outer laminal dehiscence can be observed (Figure 1(d)). If preretinal traction induces inner laminar dehiscence, a full-thickness MH may develop with a macular retinal detachment (Figure 1(e)) [44, 48].

Finally, the third pathway includes MH with concomitant chorioretinal atrophy or underlying macular scar. Both progressive retinal thinning over the lesion and tangential traction promote the MH formation that may occur adjacent to the margins or within the lesion area [44].

In a recent report, Lai et al. [49] analyzed the lamellar hole-associated epiretinal proliferation (LHEP) in lamellar MH and full-thickness MH in high myopic eyes. This epiretinal proliferation is a noncontractile membrane localized at the edge of a deep and wide retinal defect. Several OCT studies have demonstrated the tight connection between the inner retinal tissue and LHEP [50] characterized by a homogeneous mound at OCT examination [51]. Two morphological LHEP findings, typical of highly myopic eyes, were described: the broad LHEP extension and LHEP-posterior hyaloid (PH) adhesion. In eyes with high myopia, a broader extension of LHEP (>1.500 *μ*m from the foveal center) was observed when compared with nonhighly myopic patients. Moreover, a greater prevalence of LHEP-PH adhesion in eyes with high myopia was detected. The LHEP-PH adhesion was described as the growth of the LHEP along the PH surface when the PH remains attached to the margins of the MH and to the space between the hole edge and the hyaloid.

Although several mechanisms of MH formation are clearly described in the literature, the exact evolution of this retinal disease in myopic eyes is not always predictable and rapid changes from a typical configuration to another pattern may be observed [44].

Figure 1 shows different myopic macular hole patterns.

## 3. Surgical Techniques

Since the introduction of three-port PPV with gas tamponade in the 90s [11, 12], several surgical strategies have been developed for the MH repair in high myopic eyes, including silicone oil tamponade, episcleral posterior buckling, suprachoroidal buckling, and scleral shortening technique [52–55].

Although PPV with ILM peeling and intraocular gas tamponade leads to an anatomical closure rate more than 90% for idiopathic MH [56], in highly myopic eye the successful closure of MH varies considerably from 60% to 100% and some patients require multiple treatments in order to achieve the closure of the hole [20, 57]. The aim of vitrectomy is to release all vitreoretinal tractions from the vitreous and the epiretinal tissue and to reduce macular stretching caused by the ILM in order to promote retinal elongation and adaptation to the posterior staphyloma, if present.

In highly myopic eyes, the ILM appears thin, sticky, and strongly adhered to the retinal surface; moreover, preretinal membranes showed a multilayered and heterogeneous aspect composed of vitreous cortex residuals, ERM, and ILM [58]. Because of these features, ILM tends to break into several little sections rather than peel off in a sheet during its surgical removal [59]. This aspect may lead to difficult and dangerous ILM peeling because the subsequent ILM regrasping is associated with a higher risk of iatrogenic retinal breaks [60].

To avoid these difficulties, several tools have been introduced in vitreoretinal surgery for the treatment of myopic MH helping the surgeons to perform a safe and effective procedure. The chromovitrectomy helps to better identify the vitreous and the epiretinal membranes in myopic eyes. The use of triamcinolone acetonide increases the visualization of the vitreous body and vitreous cortex that remains tightly adhered to the retina plane in high myopic patients. Furthermore, the introduction of vital dyes allows to stain the posterior pole and correctly recognized the ERM (trypan blue) and the ILM (brilliant blue, lutein/zeaxanthin + brilliant blue). Moreover, the use of specific light filters can provide a better visualization of stained ILM in myopic eyes [61], reducing the retina light toxicity [62].

In order to avoid the inadvertent migration of vital dyes into the subretinal space, several substances have been used during the dye-assisted ILM peeling in MH surgery [63–65]. To promote the gentle injections of vital dyes and heavy liquids into the vitreous cavity and to avoid fluid turbulence, several devices with a steady and slow jet have been introduced [66]. Specific dual-bore cannula with a side port for fluid injection prevents the risk of retinal injury caused by a fluid jet stream and promotes a faster pressure relief during injection by multiple fluid egress vents [67].

The microscope-integrated intraoperative OCT in MH surgery allows a real-time visualization of the vitreoretinal interface [68]. This device provides an imaging of foveal architecture during the tissue-instruments interaction helping the surgeon to release in a safe manner the VMT and to identify and remove residual ERM or ILM if present [69].

In high myopic MH, several factors may influence the anatomical and functional outcomes after surgery [44] including the increased axial length, the retinal shortening over an elongated sclera [57], the presence of posterior staphyloma [70], the presence of severe traction maculopathy [63], the presence of foveoschisis [41], and the intraoperative peeling of rigid and stiff ILM [71].

The extension of ILM peeling is a matter of controversy. For a successful MH closure, no clear guidelines regarding the minimum area of ILM peeling are available [72]. Due to the adhesion between the ILM and the retinal nerve fiber layer—ganglion cell layer complex, the peeling maneuver might damage these inner retinal layers with consequently poor postoperative visual acuity [73]. Thus, a smaller ILM peeling might be more respectful of the retinal nerve fiber layer—ganglion cell layer complex [74]. On the other hand, a wider peeling extended up to the vascular arcades might be useful in order to reduce the complex tractional forces in the myopic retina [75].

However, ILM peeling in myopic FS is currently under debate. Due to thin central foveal tissue, an iatrogenic full-thickness MH could develop during ILM removal. The risk of this complication ranges from 16.7 to 20.8% [76] and significantly increases in case of outer lamellar macular hole [77]. To reduce the risk of this complication and to preserve the epifoveal ILM, foveal-sparing ILM peeling procedures were introduced [76–79]. Although these techniques have showed a better success rate and visual outcome when compared to the classic ILM peeling, no consensus regarding the ILM area to be preserve was developed. A range from 300 to 1000 *μ*m diameter was reported in different studies [77]. Anatomical results showed the primary success rate limited to 50–73.3% with vitrectomy, ILM peeling, and gas tamponade in high myopic eyes [80].

Recently, for persistent myopic MH, the inverted ILM flap technique was introduced [81]. In this procedure, the remnant of an incomplete ILM peeling is left and then inverted inside the macular hole to provide a scaffold for MH closure [81]. At the beginning, many surgeons believed necessary to insert the flap into the hole like a plug. Now it is thought sufficient to cover the hole with the peeled ILM flap in order to obtain a division between the vitreous cavity and the intraretinal spaces and to promote the MH repair mechanism [82]. The original procedure consisted of circumferentially 2-disc-diameter ILM peeling around the MH to preserve the adhesion between the inner ILM outline and the MH edges. Afterwards, the ILM flap was folded and gently positioned inside the MH [81]. For ILM flap manipulation and positioning, different techniques have been described to prevent the flap dislocation during the fluid-air exchange or in the postoperative period. These strategies include the use of viscoelastic [83] or perfluorocarbon [84] before balanced saline solution-air exchange, fluid-air exchange with low intraocular pressure and passive aspiration [75], and the addition of autologous serum [14, 22] or platelet-rich plasma [85]. Platelet-rich plasma seems to achieve better anatomical results compared to the autologous serum due to its greater adhesiveness to the edges of the hole [82].

In order to increase the ILM flap maneuverability and to reduce its displacement, several studies suggested the use of a bigger ILM flap which is resized and trimmed using the vitrectomy probe and subsequently plugged into the hole [20, 75, 86]. If ERM is present, it may be inverted with the ILM into the hole [20]. To avoid macular hole packing with a folded multilayered ILM, the creation of a single-layer inverted flap was suggested [84]. This large monolayer ILM flap seemed to promote regular glial cells proliferation and to create a physiological scaffold for the photoreceptors [84, 87]. To preserve the nasal maculopapillary nerve fiber layer, a 270-degree C-shaped temporal ILM flap has been suggested [23]. ILM flap is peeled towards the hole but stopped approximately 0.5 disc diameter from the center of the fovea, and a single smooth layer is used to cover the MH. This technique provides a reduction in dissociated optic nerve fiber layer [88] and is able to release more than 180° traction from the temporal side. A surgical variant of 180° superior ILM flap which does not require postoperatively prone positioning was also proposed [89].

The inverted ILM flap approach remains technically challenging even for an experienced surgeon [90]. To further steady the flap in an adequate position, the vitreous cavity can be filled with gas or silicone oil [20, 26, 75]. Compared to gas, silicone oil is characterized by long-lasting but weaker tamponade action, especially for myopic eyes with deep posterior staphyloma because of its difficulty to fit into this area. Nevertheless, silicone oil has been tested as a tamponade in highly myopic eyes, and it surprisingly demonstrated to be an effective tamponade for the treatment of MH [53]. It has been demonstrated that silicone oil creates a bursa inside the MH with a compartmentalization of chemotactic and nutrient substances. These substances might promote reparative mechanism which supports the closure of the hole [82].

Although the anatomic success of inverted ILM flap technique without retinal detachment range from 94% to 100% [75, 81], a previous PPV with complete ILM peeling precludes the feasibility of this surgical approach in case of MH reopening. In these cases, several alternatives have been described including the use of anterior and posterior lens capsular flap transplantation [91] or the autologous transplantation of the ILM [25]. Recently, autologous neurosensory retinal free flap transplantation (ANRFF) technique with gas or silicone oil tamponade was introduced [92, 93]. Chen et al. [91] described the use of anterior and posterior capsule lens to close refractory MH, due to its similar nature to ILM, reporting a significantly better closure rate with the anterior capsule flap but not a significantly VA improvement than eyes treated with the posterior one. Once obtained a circular anterior or posterior capsular flap, it is stained with 0.125% ICG and transplanted into the hole [91]. The advantages of a lens capsular flap are its higher density compared with ILM making it easier to settle down on the retinal surface and its easy feasibility.

Autologous transplantation of the ILM was first described by Morizane et al. [25]. This surgical approach consists of visualization of residual ILM using 0.25 mg/mL brilliant blue G solution and creation and transplantation of a free flap of ILM inside the macular hole. Grewal and Mahmoud [92] described the use of ANRFF transplantation for refractory myopic MH complicated by retinal detachment. This technique involves harvesting an autologous neurosensory retinal free flap and gently positioning it over the refractory MH using bimanual approach [92].

In highly myopic eyes, a macular hole with retinal detachment (MHRD) can occur. Vitrectomy with removal of cortical vitreous and ILM and gas or silicone oil tamponade has become the standard of care for MHRD treatment [94]. Although anatomic reattachment can be achieved, the success rate of MH closure ranges from 50% to 70% and poor postoperatively visual acuity was reported [23, 95, 96]. The inverted ILM flap technique has been demonstrated to be a useful strategy to increase the closure rate of the hole in MHRD [20, 22, 23]. Macular buckling has also been introduced as a treatment option for both primary MHRD and MHRD refractory to vitrectomy with an anatomical success rate ranging from 70% to 100% [97–101]. This technique aims to reduce the complex anteroposterior and tangential tractions resulting from PS in highly myopic eyes through the modification of macular shape from concave to convex profile. According to the intraoperative difficulty to check the correct position of the buckle and to place sutures posteriorly, several buckles with different designs and material have been described [102–104].

Considering that eyes with extremely high myopia can frequently exceed 30 mm in the axial length, surgical instrumentation, such as vitreous forceps, must be evaluated accurately during PPV. To overcome the inability to reach the retinal surface, surgeons can verticalize the instruments and carefully apply a pressure on the sclera wall in order to temporarily reduce the axial length of the globe [105]. However, these maneuvers are not free from complications, such as limited visibility due to erroneous contact and dislocation of the viewing system, corneal folds and impaired surgeon maneuverability which result in a longer operation time and challenging surgical procedures [106]. To prevent complications resulting from iatrogenic trauma, long-shaft forceps for ILM peeling specific for highly myopic eyes were developed [107]. Curved elongated instruments were also proposed [105].

Microincision vitrectomy surgery (MIVS) has potential advantages compared to conventional vitrectomy in highly myopic eyes. Despite the lowest incidence of complications with the small-gauge system compared to conventional vitrectomy, no statistically significant difference in visual outcomes has been reported [59]. With MIVS a small-diameter sutureless sclerotomies may be performed. This surgery is characterized by a faster postoperative recovery, less postoperative inflammation and patient discomfort, and reduction of iatrogenic peripheral breaks incidence [108, 109]. However, in some cases, the self-healing sclerotomies can remain open with spontaneous leakage and early postoperative hypotony. This condition mainly occurs in myopic patients due to the anatomical characteristic of these eyes with a thinner sclera and a deranged fibrillar architecture [110].

The intrascleral tunnel morphology plays a key role in avoiding the sclerotomy leakage. Both biplanar oblique-perpendicular-bevel-down trocar insertion and longer tunnel have a lower incidence of early hypotony when compared to straight bevel-up and shorter sclerotomies [111–114]. To promote the wound sealing, several options have been introduced, including the prolonged sclerotomy massage [115, 116], transconjunctival and trans-scleral absorbable suture [117], releasable sutures [118], use of fibrin glue or polyethylene glycol hydrogel polymer [119, 120], and conjunctival cauterization [117]. The introduction of 27-gauge vitrectomy with scleral incision diameter of 0.4 mm does not require biplanar trocar insertion, thus reducing the risk of postoperative hypotony in myopic eyes [121, 122].

## 4. Conclusions

Several and complex tractional mechanisms are implicated in the development of MH in myopic eyes including the anteroposterior forces due to the strong adhesion of vitreous cortex, the tangential traction on the macula caused by the ILM, as well as the centripetal vector forces exerted by the PS. Related to these anatomical conditions, myopic eyes still remain a challenge for vitreoretinal surgeons. In the last decades, the introduction of significant innovations for the treatment of myopic MH has allowed vitreoretinal surgeons to perform a safe and effective surgery in these complicated eyes.
